# 基于雌激素受体信号通路的肺癌靶向治疗研究进展

**DOI:** 10.3779/j.issn.1009-3419.2011.09.07

**Published:** 2011-09-20

**Authors:** 利强 徐, 永德 廖, 和孝 唐, 超 张, 昭国 刘

**Affiliations:** 1 430030 武汉，华中科技大学同济医学院附属同济医院普胸外科 Department of General Thoracic Surgery, Tongji Hospital, Tongji Medical College, Huazhong University of Science and Technology, Wuhan 430030, China; 2 442000 十堰，湖北医药学院附属太和医院胸心外科 Department of Cardiothoracic Surgery, Taihe Hospital, Hubei University of Medicine, Shiyan 442000, China

**Keywords:** 雌激素受体, 肺肿瘤, 靶向治疗, Estrogen receptor, Lung neoplasms, Targeted therapy

## Abstract

越来越多的证据表明，雌激素对多种肿瘤的发生发展有促进作用，不仅包括雌激素靶器官肿瘤，还包括非靶器官肿瘤。雌激素通过两种不同的雌激素受体（estrogen receptor, ER）亚型——ERα和ERβ来发挥其调节作用，介导细胞增殖和分化。近几十年来，随着ERα介导的信号通路在乳腺癌中作用的阐明，针对ER信号的靶向治疗已经成功地应用于临床，其经典药物他莫昔芬是一种选择性雌激素受体调节剂（selective estrogen receptor modulator, SERM）。随着雌激素在肺癌恶性进展中的作用逐渐为人们所认识，针对ER信号通路的靶向治疗在肺癌中的应用也逐渐受到重视，并将有可能成为肺癌综合治疗的重要组成部分。

## Abstract (en)

Increasing evidence indicates that estrogen promotes tumor growth in both estrogen target organs and non-target organs. Estrogen regulates cell proliferation and differentiation via two different receptors, estrogen receptors α and β (ERα and ERβ). In recent decades, with the clarification of the ERα-mediated signaling pathways in breast cancer, targeted therapy through these pathways have successfully been used in clinical application. Tamoxifen, the classic representative, is a selective estrogen receptor modulator (SERM). Along with the elucidation of the role of estrogen in the pathophysiology of lung cancer, targeted lung cancer treatment based on the ER signaling pathways is also gradually being applied and it could become an important part of the comprehensive treatment for lung cancer.

## 1 前言

目前肺癌已成为男性和女性恶性肿瘤最常见的死因之一^[1, 2]^。虽然采取了手术、放化疗和靶向治疗的综合治疗策略，肺癌的生存率仍较低^[3]^。因此，寻找新的肺癌治疗切入点显得尤为迫切。虽然肺癌的病因和机制尚不清楚，但是不断发现的证据^[4-9]^表明：雌激素是促进肺癌特别是女性肺腺癌进展的因素之一。抗雌激素治疗或者针对雌激素受体（estrogen receptor, ER）信号的靶向治疗在肺癌中的应用可能有助于改善患者的预后。

## 2 雌激素与肺癌

流行病学研究^[10, 11]^表明，吸烟仍然是肺癌最主要的危险因素，但在同等吸烟水平下，女性比男性患肺癌的风险更大；在不吸烟的肺癌患者中，女性也占多数，女性患肺癌的风险较男性高2.5倍。在绝经后女性中进行的激素替代治疗（hormone replacement therapy, HRT）研究^[12-15]^发现，行HRT的绝经后妇女发生肺癌的风险增高，而已患肺癌者生存期较对照组缩短。结合肺癌发生率的性别差异以及外源性雌激素对女性肺癌进展的促进作用，再考虑到女性体内本身存在的较高循环血雌激素水平，可以推测高雌激素水平可能使得女性对肺癌更加易感^[16]^。目前，随着女性吸烟人数的增加，美国每年死于肺癌的女性患者仅次于乳腺癌，居第二位^[1]^。

虽然男性肺癌患者体内循环血雌激素水平较女性低，但是在非小细胞肺癌（non-small cell lung cancer, NSCLC）内却检测到较高的雌激素水平，较良性病变中高2.2倍；特别是男性NSCLC，其局部雌激素浓度是绝经后女性的3.7倍^[7, 17]^。这些研究结果均提示雌激素可能也与男性肺癌密切相关。

雌激素的促肺癌进展作用在NSCLC的细胞系和动物模型中已经得到许多研究的支持。雌激素与NSCLC细胞系共培养，能刺激细胞产生明显的增殖，而抗雌激素药物如他莫昔芬或氟维司群能阻断这种效应^[6, 8, 11, 18-20]^。虽然有研究^[21-23]^在男性肺腺癌细胞中观察到不同的结果，但是在将NSCLC细胞植入小鼠皮下形成的移植瘤模型中，雌激素仍显示出促进肿瘤生长的作用，抗雌激素药物处理可以明显抑制肿瘤生长^[6, 11, 19, 20]^。这些研究表明，雌激素及其启动的ER信号通路可以促进肺癌进展。因此，抗雌激素或ER信号的靶向治疗策略可能是肺癌治疗的新切入点。

## 3 ER结构和ER信号通路

雌激素经肿瘤局部芳香化酶合成后，主要经ER发挥作用。ER的两种亚型——ERα和ERβ由不同染色体上的不同基因编码，分别位于6q24区段和14q22-24区段。ER属于核受体超家族成员，由6个保守的结构域（A-F）组成，主要包括3个功能区：转录激活区（transcriptional activation function, TAF）、DNA结合区（DNA binding domain, DBD）和配体结合区（ligand binding domain, LBD）。位于C域中央的DNA结合区含有两个高度保守的锌指结构，ER通过此区与靶基因启动子中的雌激素反应组件（estrogen response element, ERE）结合，调节基因转录。

在肺癌的ER信号通路中，雌激素与ER结合后主要通过非基因途径和基因途径产生效应。其中非基因途径的特点是由细胞内信号分子如MAPK、Akt等通过蛋白之间的作用快速启动，并引起相关信号分子的磷酸化，这些产物一般在数分钟左右作用达峰值^[19, 24, 25]^；基因途径主要由ERE介导的经典途径和以AP-1（activator protein 1, AP-1）等为代表的转录因子（non-ERE）介导的非经典途径组成^[26, 27]^。在经典途径中，活化的ER形成二聚体直接与靶基因启动子中的ERE结合，在共调节因子的参与下调节基因转录。但在非经典基因途径中，活化的ER形成二聚体后不与ERE结合，而是通过蛋白间相互作用与Fos/jun等形成复合体，再与其它转录因子如AP-1结合，在共调节因子的参与下调节基因转录^[26]^。

## 4 基于ER信号的肺癌靶向治疗策略

ER介导的上述信号通路中，关键步骤包括局部环境雌激素的生成、雌激素与ER的结合、ER二聚体的形成、与DNA的结合以及共活化物的募集。在肺癌中，阻断其中任何步骤，都可以阻断或干扰ER信号的传导，从而抑制其介导的肿瘤发展。因此，这些环节都可能成为肺癌潜在的治疗靶点。

### 4.1 芳香化酶抑制剂（aromatase inhibitor, AI）

芳香化酶属于细胞色素P450酶系，是雌激素合成过程中最后一步的限速酶。其不仅表达于卵巢，还表达于其它组织，如乳房、肺、肝脏和脑组织等^[28]^。多项研究^[29-31]^表明在不同性别的NSCLC组织标本中都发现芳香化酶高表达。进一步研究^[7, 29]^发现，在86%的NSCLC肿瘤组织中，雌激素浓度和芳香化酶表达同时增加，且芳香化酶有较高的活性，与癌旁和良性组织中表达差异明显。这些研究都提示，NSCLC肿瘤中雌激素的高表达可能与局部芳香化酶介导的雌激素合成有关。绝经后女性NSCLC患者中，高表达芳香化酶提示预后不良^[30]^，这可能是由局部高浓度的雌激素持续刺激肿瘤生长导致的^[31, 32]^。

而AI则可以阻断雌激素的合成，从而降低局部雌激素浓度，消除雌激素对肿瘤的促进作用。由此推测，高表达芳香化酶的NSCLC患者可能从AI治疗中获益^[20, 31, 32]^。目前的第三代芳香化酶抑制剂分为两类，包括可逆性非甾体类抑制剂（如阿那曲唑和来曲唑）和不可逆性甾体灭活剂（如依西美坦）。

Coombes等^[33]^报道，在随机分组的原发性乳腺癌患者中，一组使用他莫昔芬2年-3年后改为芳香化酶抑制剂依西美坦，另一组则一直使用他莫昔芬，数年后发现改用依西美坦的患者患原发性肺癌的危险性下降（使用他莫昔芬2年-3年后改为依西美坦组肺癌患者4例*vs*一直使用他莫昔芬组肺癌患者12例）。Weinberg等^[29]^在临床前研究中发现，阿那曲唑不仅可以明显抑制体外培养的NSCLC细胞增殖，在体内实验中也有明显的抑制瘤体生长的作用。Koutras等^[34]^在对NSCLC细胞系的研究中发现，依西美坦能够抑制癌细胞增殖并促进凋亡。Marquez等^[20]^在裸鼠的NSCLC中也验证了AI的抗肿瘤作用，同时还发现AI与化疗联用可以对肿瘤进展产生明显的协同抑制作用。更重要的是，研究证实局部转移的癌组织高表达芳香化酶，这提示晚期NSCLC患者的远处转移灶也可从AI治疗中获益。

以上这些研究结果从作用及机制方面对芳香化酶进行了较为深入的研究，是芳香化酶抑制剂用于临床肺癌治疗的重要研究基础和实验依据；并且后续的芳香化酶药物的开发也是重要的尝试，以期在芳香化酶抑制剂治疗肺癌方面有所突破。目前，在患晚期NSCLC的绝经后妇女中进行的一项为期18个月的临床实验（编号：NCT00932152）中，包括芳香化酶抑制剂阿那曲唑（1 mg/d，口服）在内的抗雌激素药物已被用来研究其对肿瘤进展的抑制作用。这些研究都表明，AI在NSCLC临床中具有良好的开发潜力。

### 4.2 选择性雌激素受体调节剂（selective estrogen receptor modulator, SERM）

SERM为ER竞争性拮抗剂，在ER阳性表达的肿瘤组织中，SERM可与ER结合但不刺激转录或作用微弱^[35]^，并长时间阻滞于ER的LBD区，干扰ER介导的信号通路，降低其介导的转录生长因子水平，从而抑制肿瘤增殖。SERM代表性药物是他莫昔芬（tamoxifen, TAM），它作为最早被批准用于乳腺癌临床治疗的SERM，目前已取得了良好的疗效。

在NSCLC细胞系的体外实验中已经证实了他莫昔芬的抗增殖作用。朱晓莉等^[36]^发现他莫昔芬和4-羟基他莫昔芬都能抑制ER(+)肺腺癌细胞株SPC-A-1的生长，并呈剂量依赖性。其主要作用机制是阻滞细胞停留于静止期，使增殖期细胞比例减少，从而抑制细胞增殖。Dougherty和Ivanova等^[21, 22]^也分别在女性来源的肺腺癌细胞中证实了TAM对细胞增殖的抑制作用。

目前，在肺癌的综合治疗中，尝试较多的SERM是TAM和托瑞米芬（toremifene, TOR）。约30%的乳腺癌患者在接受SERM治疗后发生继发性耐药^[37]^。因此，TAM在肺癌中虽然可以作为ER的拮抗剂来抑制ER信号通路，但是不单用，而多作为化疗多药耐药（multiple drug resistance, MDR）的调节剂^[38-40]^，逆转肺癌对一线顺铂类化疗药物的耐药，提高化疗敏感性，从而增加疗效。

Yang等^[41]^报道，高剂量的TAM（150 mg/m^2^/d）联合常规化疗，有助于改善晚期NSCLC患者的预后。Chen等^[39]^也发现，对一线顺铂类化疗方案耐药的NSCLC患者，在联合TAM（120 mg/d）治疗后，仍可获得较好的疗效。但是王昆等^[42]^在临床实验中发现，与对照组相比，TAM（20 mg/d）联合化疗应用于NSCLC没有明显改善患者的预后水平，这可能与TAM剂量过低、入选病例数过少等有关。Perez等^[43]^进一步研究发现，联合顺铂治疗肺癌时，口服TAM（160 mg/m^2^/d）连续7天才能达到有效的作用水平（约5 mM），从而增加肿瘤对顺铂的敏感性，并且此时TAM的副作用仍可被接受。TAM口服耐受性好，仅少数患者出现不良反应，如恶心呕吐、月经失调、颜面皮疹等。

### 4.3 选择性雌激素受体下调剂（selective estrogen receptor downregulator, SERD）

SERD为ER非竞争性调节剂，与ER的LBD区结合后，诱导蛋白酶体介导的ER快速降解，从而抑制ER信号通路，抑制肿瘤增殖。其代表药物是氟维司群（fulvestrant, ICI182780），其结构与雌激素类似，仅在7α部位多出一个长侧链。与TAM相比，由于无雌激素激动活性且具有不刺激子宫内膜增殖等优点，因此SERD被称为纯抗雌激素。它的作用机制包括以下几个方面^[44-47]^：（1）竞争性与ER结合后，促使ER快速降解；（2）抑制ER二聚体的形成及其向核内转移定位，从而阻断ER信号的基因途径；（3）抑制ER与ERE的结合，减少ERE介导的转录效应。

多项临床前研究证实了氟维司群在NSCLC细胞系和移植瘤动物模型中对肺癌进展的抑制作用。Hershberger等^[8]^发现氟维司群可以抑制男性来源的肺腺癌201T和鳞癌273T细胞系增殖，Marquez等^[11]^进一步报道，在卵巢切除后的裸鼠肺腺癌A549细胞皮下移植瘤模型中，氟维司群能够明显抑制肿瘤的生长，与厄洛替尼联用可产生明显的协同抑制效应。Stabile等^[19]^发现氟维司群不仅可以抑制NSCLC细胞系的增殖，还能有效抑制严重联合免疫缺陷（severe combined immunodeficiency disease, SCID）鼠皮下移植瘤的生长。

已经有将氟维司群用于联合吉非替尼治疗绝经女性NSCLC的临床研究，肌注给药250 mg/月耐受性良好^[48]^。目前，以患晚期NSCLC的绝经后妇女为对象正在进行的一项Ⅱ期临床实验（编号：NCT00932152）用于研究联合氟维司群（负荷量：首轮第1天500 mg，第14天250 mg；以后250 mg/月，肌注）和阿那曲唑在一线化疗后巩固治疗中的价值。这些研究从细胞和动物实验等不同角度对SERD进行了重要的研究，为临床实验积累了重要的实验依据和数据资料。在SERD治疗肺癌方面正在进行的临床实验是新的探索，它的突破将是以后肺癌治疗研究的热点之一。

### 4.4 多靶点联合治疗

值得注意的是，在肿瘤的靶向治疗中，单一靶点的治疗往往阻断不够完全，因此效果差；同时因为细胞内相关信号通路的交互作用，阻断其中一条通路又会导致其它通路的回馈性启动，进而诱导继发性耐药的发生。正如目前肺癌中表皮生长因子受体（epidermal growth factor receptor, EGFR）的靶向药物吉非替尼存在的问题一样，单药治疗不仅有效率低（10%左右），而且容易产生继发性耐药^[49]^。因此，在针对ER信号通路的肺癌靶向治疗中，与其它靶点药物联合应用是未来的发展方向，不仅可以相互协同产生更强的抗肿瘤效应，还能延迟各自耐药的发生。

#### 4.4.1 他莫昔芬或氟维司群联合EGFR抑制剂靶向治疗

在NSCLC中ER和EGFR两条信号通路之间的交互对话已经被证实^[5]^，因此联合靶向抑制两条信号可能产生协同抑制作用。在NSCLC细胞和动物模型实验中，多项研究^[11, 18, 20, 50]^已证实两种信号通路抑制剂之间的协同作用，且已经有早期临床应用的报道^[48]^。目前有多项临床实验正在进行中，以进一步评估联合氟维司群和EGFR抑制剂对晚期NSCLC的临床价值（编号：NCT00100854，NCT01004419，NCT00592007）。

#### 4.4.2 AI联合EGFR靶向治疗

研究^[51, 52]^发现在乳腺癌细胞中前列腺素E2（prostaglandin E2, PGE2）和环氧合酶-2（cyclooxgenase-2, COX-2）不仅能刺激芳香化酶表达，还能增强其活性。而在NSCLC中的前期研究^[53]^提示，表皮生长因子（epidermal growth factor, EGF）和β-转化生长因子（transforming growth factor-β, TGF-β）等能通过诱导COX-2的表达来增加PGE2的浓度。进一步研究^[20]^发现，在NSCLC中，EGFR信号可以增强芳香化酶的表达和活性。这说明EGFR和ER信号之间可能存在另一种模式的交互作用^[19, 20]^，因而联合AI和EGFR靶向药物对NSCLC的抑制可能产生协同作用。

#### 4.4.3 ER联合胰岛素样生长因子-1受体（IGF-1R）靶向治疗

前期临床研究发现：在肺癌患者外周血中，雌激素和胰岛素样生长因子1（insulin-like growth factor 1, IGF-1）水平同时升高。而且在肺癌组织中，ERβ和IGF-1、IGF-1R在同一肺癌组织中同时过表达。进一步体外研究发现，雌激素能上调IGF-1R信号通路关键分子IGF-1和磷酸化IGF-1R的表达。这些现象共同提示ER信号通路和IGF-1R信号通路在肺癌中可能存在交互作用。因此，联合ER和IGF-1R靶向药物治疗肺癌也是发展方向之一。

## 5 展望

近年来许多研究报道了ERβ在肺癌中的过表达现象^[54, 55]^以及其特异性激动剂对肺癌细胞系的促增殖作用^[24, 25, 56]^，这些都提示ERβ可能是雌激素在肺癌中起作用的主要功能受体。因此，开发针对ERβ的特异性阻断剂可能对肺癌的治疗有积极意义。针对ER与DNA结合、共活化因子的募集等ER信号通路环节的靶向药物已在研发中。目前，ER信号在肺癌发生发展中的作用机制尚不清楚，但我们有理由相信随着这一机制的深入阐明可以为基于ER信号的靶向治疗提供更多的新思路和切入点。

虽然对雌激素在肺癌进展中的促进作用已经形成了初步的共识，诸多的临床前研究也证实了针对ER信号通路的各种靶向治疗策略的有效性，但是肺癌的抗雌激素治疗在临床上应用的广泛开展和效果的最终评价尚需要雌激素促肺癌机制的进一步深入研究以及大样本前瞻性的规范研究提供支持和依据。
